# Phase I study of PD 0332991, a cyclin-dependent kinase inhibitor, administered in 3-week cycles (Schedule 2/1)

**DOI:** 10.1038/bjc.2011.177

**Published:** 2011-05-24

**Authors:** G K Schwartz, P M LoRusso, M A Dickson, S S Randolph, M N Shaik, K D Wilner, R Courtney, P J O'Dwyer

**Affiliations:** 1 Memorial Sloan-Kettering Cancer Center, 1275 York Avenue, New York, NY 10021, USA; 2 Karmanos Cancer Institute, 4100 John R St., Mail Code: HW04HO, Detroit, MI 48201, USA; 3 Pfizer Oncology, 10646 Science Center Drive, San Diego, CA 92121, USA; 4 Pfizer Oncology, 10555 Science Center Drive, San Diego, CA 92121, USA; 5 Abramson Cancer Center, University of Pennsylvania, Philadelphia, PA 19104, USA

**Keywords:** PD 0332991, CDK inhibitor, retinoblastoma-positive solid tumours, non-Hodgkin's lymphoma, G1/S checkpoint

## Abstract

**Background:**

This phase I, open-label, first-in-human study determined dose-limiting toxicities (DLTs) and maximum tolerated dose (MTD) of PD 0332991, an oral cyclin-dependent kinase 4/6 inhibitor with potent anti-proliferative activity *in vitro*/*vivo*.

**Methods:**

A total of 33 patients with retinoblastoma protein-positive advanced solid tumours or non-Hodgkin's lymphoma refractory to standard therapy or for which no therapy was available received PD 0332991 once daily (QD) for 14 days followed by 7 days off treatment (21-day cycles; Schedule 2/1).

**Results:**

Six patients had DLTs (18% four receiving 200 mg QD; two receiving 225 mg QD); the MTD was 200 mg QD. Treatment-related, non-haematological adverse events occurred in 29 patients (88%) during cycle 1 and 27 patients (82%) thereafter. Adverse events were generally mild–moderate. Of 31 evaluable patients, one with testicular cancer achieved a partial response; nine had stable disease (⩾10 cycles in three cases). PD 0332991 was slowly absorbed (mean *T*_max_ 4.2 h) and eliminated (mean half-life 26.7 h). Volume of distribution was large (mean 3241 l) with dose-proportional exposure. Using a maximum effective concentration model, neutropenia was proportional to exposure.

**Conclusion:**

PD 0332991 was generally well tolerated, with DLTs related mainly to myelosuppression. The MTD, 200 mg QD, is recommended for phase II study.

Progression through the cell cycle, which is fundamental to cell proliferation, is controlled by the sequential activation of a family of related cyclin-dependent kinases (CDKs). Cyclin-dependent kinase 4 and 6 (CDK4/6) are closely related kinases that, together with their activating subunits, the D-type cyclins, promote cell-cycle progression from G1 to S phase via phosphorylation of the retinoblastoma (Rb) protein (Dickson and Schwartz, 2009).

Most tumours circumvent control of G1 to S progression using a variety of mechanisms. In a minority of tumours (such as Rb and small-cell lung cancer), control is lost through mutation of the *Rb* gene (Friend *et al*, 1986; Harbour *et al*, 1988). In the majority of cases, however, other genetic and epigenetic changes lead to increases in CDK4/6 activity contributing to tumour cell growth (Shapiro, 2006). Tumour cell proliferation may therefore be particularly sensitive to selective inhibition of CDK4/6.

PD 0332991 is an orally administered, highly specific reversible inhibitor of CDK4/6 (Fry *et al*, 2004; Toogood *et al*, 2005). PD 0332991 has a potent anti-proliferative effect *in vitro*, leading to cell-cycle arrest at G1, but has no activity against Rb-negative cell lines (Fry *et al*, 2004). Similarly, PD 0332991 significantly reduces tumour growth and leads to tumour regression in several Rb-positive human xenograft models *in vivo*, without activity against Rb-negative tumours (Fry *et al*, 2004).

Results from the first-in-human phase I study of PD 0332991 on a schedule of 2 weeks on treatment followed by 1 week off treatment (Schedule 2/1) are reported here. This 3-week schedule allows combination with other cancer therapies that are administered using a 3-week dosing cycle.

## Materials and methods

### Study design

This was a phase I, dose-finding, open-label, non-comparative study of PD 0332991 in patients with Rb-positive solid tumours or non-Hodgkin's lymphoma (NHL). As pre-clinical data suggest that tumours that do not express Rb will not respond to treatment with PD 0332991, histologically proven Rb tumour expression was an essential eligibility criterion for patients entering this study. On the basis of the low frequency of Rb expression in small-cell lung cancer and Rb (Lee *et al*, 1987; Yokota *et al*, 1988; Shimizu *et al*, 1994), patients with both tumour types were excluded from the trial.

The primary objectives of this study were to establish the safety profile of PD 0332991 given in a 3-week treatment cycle by identifying dose-limiting toxicities (DLTs), the maximum administered dose and the maximum tolerated dose (MTD), and to establish the recommended dose for phase II studies (RP2D). Secondary objectives included characterisation of single-dose and steady-state pharmacokinetics (PKs) of oral PD 0332991 and evaluation of preliminary antitumour activity.

### Study population

Eligible patients were men and women aged ⩾18 years with histologically or cytologically confirmed Rb-positive solid tumours (except small-cell lung cancer or Rb) or NHL refractory to standard therapy, or for whom no standard-of-care therapy was available. Retinoblastoma expression was assayed by immunohistochemistry at a central laboratory using an archival or fresh tumour biopsy; eligibility required level ⩾1+ staining (i.e., of at least the minimal intensity considered above background level). Additional eligibility criteria included Eastern Cooperative Oncology Group (ECOG) performance status ⩽2, the ability to swallow intact PD 0332991 capsules, and adequate organ and haematological function, including haemoglobin >9.0 g dl^−1^ without transfusions (erythropoietin to be discontinued ⩾2 weeks before the first dose of study medication).

Patients were ineligible, if they met any of the following criteria: cytotoxic chemotherapy within 3 weeks before first treatment (8 weeks for mitomycin C or nitrosoureas); hormone therapy, radioimmunotherapy, immunotherapy or other biological therapy within 14 days before treatment; QTc interval >470 msec; and untreated brain metastases. Patients with brain metastases were eligible, if they had completed treatment ⩾10 days before starting study medication, had discontinued corticosteroid treatment for these metastases for ⩾5 days and were neurologically stable.

The final study protocol and amendments were reviewed and approved by the Institutional Review Board/Independent Ethics Committee at each participating site, in accordance with the International Conference on Harmonization Good Clinical Practice guidelines and the Declaration of Helsinki. Written informed consent was obtained from all patients before study participation.

### Treatment

Cohorts of patients received escalating doses of PD 0332991 given daily for 2 weeks, followed by 1 week off treatment (Schedule 2/1). Treatment cycles were repeated until the occurrence of disease progression, unacceptable toxicity or withdrawal of consent.

Three to four evaluable patients were initially enrolled per dose-escalation cohort, and the cohort expanded to six evaluable patients, if a DLT occurred during the first treatment cycle. If two DLTs were observed in a single cohort during the first treatment cycle, dose escalation was stopped, and dose finding was continued at a lower level. The MTD was defined as the highest dose level for which the incidence of first-cycle DLT was <2/6. Cohorts receiving the MTD were expanded to include a minimum of 12 patients. If <33% of patients in the MTD cohort had a DLT, then the dose taken by this cohort was considered to be the RP2D.

Dose-limiting toxicity was defined as one of the following adverse events (AEs) occurring during cycle 1: grade 4 haematological toxicity; grade 3 neutropenia with infection or fever ⩾38.5°C; grade ⩾3 non-haematological treatment-related toxicity, except toxicities which had not been maximally treated (such as nausea, vomiting and diarrhoea) or that the patient considered tolerable (such as skin rash); confirmed grade 3 QTc prolongation (QTc >500 ms) persisting after correction of other possible causes such as electrolyte imbalance or hypoxia; or inability to receive the next dose of PD 0332991 within 1 week (±1 day) of the last dose because of lack of haematological recovery or prolonged non-haematological toxicity grade ⩾3. The dose for patients resuming treatment following a DLT was reduced to the next lower dose level or by 50%, if the DLT occurred at the starting dose of the trial.

### Safety assessments

Safety was assessed at baseline, at regular intervals throughout the study, and within 1 week following treatment discontinuation by recording AEs, haematological and biochemical parameters, and physical status (including ECOG performance status). Adverse event severity was graded using the National Cancer Institute Common Terminology Criteria for Adverse Events, version 3.0 (Trotti *et al*, 2003). Triplicate electrocardiograms (ECGs) ∼2 min apart were taken at baseline, 3 h post-dose on days 1, 8 and 15 of cycle 1, and on day 15 of subsequent cycles for up to six cycles.

### Tumour response

Tumour measurements derived from computed tomography or magnetic resonance imaging scans were obtained at baseline, after every 2 cycles during the study, and at the end of treatment. Tumour responses were evaluated based on Response Evaluation Criteria in Solid Tumors, version 1.0 (Therasse *et al*, 2000).

### Pharmacokinetic assessments

Samples for measurement of PK parameters were collected from all patients pre-dose and at intervals up to 10 h post-dose following a single administration (cycle one, day 1) and after repeated dosing (cycle 1, day 8). In cycle 1, three additional blood samples for PK analysis were collected during the 7-day interval without treatment on days 15, 16 and 17. In addition a sample was collected from patients in the expanded MTD cohort 24 h post-dose on cycle 1, day 1. Urine PK assessments (0–10 h post-dose) were carried out on cycle 1, day 1 in patients in the expanded cohort. Blood draws for PK assessment were also obtained at the same time as ECG testing at the projected time-to-first occurrence of the maximum observed plasma concentration (*C*_max_). Individual area under the concentration–time curve (AUC) values estimated based on dose and individual apparent clearance (CL/F) was obtained from population PK modelling. Additional PK parameters assessed were the time-to-first occurrence of *C*_max_ (*T*_max_), the apparent volume of distribution during the terminal phase (V_z_/F), the terminal elimination half-life (*t*_1/2_) and the drug accumulation ratio (*R*_ac_).

### Pharmacodynamic assessments

To document the course of changes in absolute neutrophil count (ANC) and platelets over time during the first two cycles of treatment, an exploratory analysis of data available from the weekly safety laboratory tests was carried out. Possible relationships between PD 0332991 exposure and changes in (i) ANC and (ii) platelets were assessed further by fitting a simple model (without baseline) to determine the maximum effective concentration (*E*_max_). Parameters determined were the *E*_max_ for ANC or platelets as a function of PD 0332991 concentration and the EC_50_ (the estimated plasma concentration resulting in a change from baseline of 50% in ANC or platelets). Data from all patients receiving PD 0332991 on Schedule 2/1 were included in the exploratory and modelling analyses.

### Statistical methods

Pharmacokinetics parameters were determined using non-compartmental analyses (WinNonLin version 4.1; Pharsight, Mountain View, CA, USA). Descriptive statistics for PK parameters were summarised by dosing group, day and cycle.

## Results

### Patient characteristics and disposition

A total of 33 patients were enrolled across four cohorts receiving the following daily doses of PD 0332991: 100 mg (*n*=3), 150 mg (*n*=4), 200 mg (*n*=20) and 225 mg (*n*=6). Patient baseline characteristics are shown in Table 1. Overall, patients received a median of two cycles of treatment (range 1–33). The median relative dose intensity in each cohort was 1.0 (range 0.29–1.04), with a total cumulative dose of PD 0332991 administered of 12 013 mg m^−2^ (s.d. 16 324 mg m^−2^). Relative dose intensity was defined as the cumulative dose (mg per week) from treatment start till the end of the treatment divided by the planned cumulative dose, where the end of the treatment was the start date of the last cycle plus the planned cycle duration.

At the time of analysis, treatment was ongoing in three patients, and the remaining 30 patients had discontinued because of AEs (*n*=2; none was considered to be treatment related), death (*n*=1), refusal of further treatment (*n*=1), progressive disease (*n*=25) or other reasons (*n*=1).

### Determination of MTD

The sequence of dose escalation progressed from 100 to 150 mg once daily (QD), and then to 225 mg QD, which was the maximum administered dose. The dose was then reduced to 200 mg QD. No DLT was observed in the first two cohorts. In the 225-mg QD cohort, two of six patients experienced DLTs. These included a patient who experienced grade 4 neutropenia and thrombocytopenia, and a patient with grade 3 neutropenia, such that the initiation of cycle 2 dosing was delayed, thereby constituting a DLT. The incidence of neutropenia reported as AEs during cycle 1 is presented in Table 2. Additional key toxicities reported in the patients in the 225 mg per day cohort experiencing DLTs included grade 1 fatigue and diarrhoea in the patient with neutropenia as the DLT, and grade 1 diarrhoea and grade 2 anaemia in the patient who had neutropenia plus thrombocytopenia.

Because of the DLTs at 225 mg QD, an intermediate dose of 200 mg QD was selected for evaluation. With only one DLT (grade 3 neutropenia/grade 3 thrombocytopenia resulting in cycle 2 delay) among six patients treated at 200 mg QD, this dose level was selected as the MTD and was expanded to include a total of 20 patients for further evaluation of safety, efficacy and biomarkers. An additional three patients in the expanded cohort experienced DLTs, comprising grade 3 neutropenia resulting in cycle two delay (*n*=2) and grade 3 neutropenia with grade 3 thrombocytopenia, also resulting in cycle 2 delay (*n*=1). The four DLTs in the 20-patient expanded cohort were considered acceptable, as the DLT rate of 20% was well within the pre-specified upper limit of 33% used to determine the MTD in the initial dose-escalation cohort.

### Overall safety

Treatment was generally well tolerated. No patient discontinued treatment permanently because of treatment-related AEs. Five patients required dose reductions, including three patients with haematological AEs associated with DLTs (two patients receiving 250 mg QD and one receiving 200 mg QD) and two patients with gastrointestinal AEs (one patient with grade 2 nausea and one patient with grade 2 vomiting and headache, both receiving 200 mg QD). In addition, treatment was interrupted following treatment-related AEs in eight patients receiving 200 mg QD during cycle 1 and in three patients after cycle 1 (two from the 200-mg QD cohort and one in the 225-mg QD cohort).

Treatment-related non-haematological AEs were observed in 29 of 33 patients (88%) across all cohorts during cycle one and in 27 of 33 patients (82%) after cycle one. These AEs were generally mild to moderate in severity, with no treatment-related grade 4 non-haematological AEs, no serious treatment-related AEs and no grade 5 treatment-related AEs observed. Treatment-related AEs occurring in ⩾5% of patients are presented in Table 3. The most common non-haematological AEs reported during cycle 1 were fatigue (33%), nausea (30%), diarrhoea (18%), constipation and rash (each 12%); after cycle 1, the most frequently reported AEs comprised diarrhoea and fatigue (each 30%), nausea (15%) and epistaxis (12%). Five grade 3 non-haematological AEs were reported (*n*=1 each), all of which occurred after the first cycle and comprised fatigue, hyperglycaemia, hyponatremia, nausea and vomiting.

Haematological toxicity was common (Table 4). Grade 3 or 4 haematological toxicity consisted of lymphopenia (36%), neutropenia (24%), leukopenia (21%), thrombocytopenia (9%) and anaemia seen in a single patient (3%).

Analysis of QT interval changes using Bazett's and Fridericia's corrections showed that Fridericia's was the more appropriate correction method based on plots of the QTc *vs* RR interval (data not shown). Using Fridericia's correction, no patients had a maximum on-treatment value ⩾500-msec or a >30-msec increase from baseline.

### Tumour response

Of the total 33 patients, 31 completed at least one post-treatment tumour assessment and were considered evaluable for response. One patient with testicular cancer had a partial response (PR) with PD 0332991 administered at 200 mg QD on Schedule 2/1. This patient had non-seminomatous germ cell tumour and had previously been treated with bleomycin, cisplatin and etoposide in the adjuvant setting. An additional nine patients (29%) experienced stable disease (SD) lasting ⩾2 cycles. Stable disease was observed in the following tumour types: liposarcoma (*n*=4), thyroid (*n*=1), melanoma (*n*=1), cholangiosarcoma (*n*=1) and angiomyxoma (*n*=1). Six of the patients (19%) with SD had SD lasting ⩾4 cycles, and three patients (10%) had SD for ⩾10 cycles. Stable disease lasting ⩾10 cycles was observed in two patients with liposarcoma (one in the 100-mg QD cohort lasting 11 cycles and one in the 225-mg QD cohort lasting ⩾23 cycles) and one patient with angiomyxoma in the 200-mg QD cohort lasting 19 cycles. The course of patients with liposarcoma can be associated with prolonged SD. However, the patient with liposarcoma, who remains on study with SD after 23 cycles, is particularly noteworthy, having progressed previously on a phase II clinical trial with sorafenib. In addition, this patient continues on trial without cumulative toxicity. Two patients were on study at the time of data cutoff: the patient with testicular cancer who achieved a PR and the patient with liposarcoma and SD lasting ⩾23 cycles.

### Pharmacokinetics

On day 1 of treatment, PD 0332991 was detectable in the plasma of all patients at the first measured timepoint (i.e., 1 h post-dose). Exposure (AUC_(0–10)_ and *C*_max_) increased in a broadly dose-proportional manner over the range of 100–225 mg PD 0332991 on days 1 and 8, although there was some variability particularly at the 150-mg dose level (Table 5, Supplementary Figures 1A and B). Overall, values for C_max_ and AUC_(0–10)_ showed low to moderate inter-patient variability after both single- and multiple-dose administration. Plasma PK parameters showed low to moderate variability with a generally dose-dependent increase in exposure over the dose range of 100–225 mg based on *C*_max_ and AUC_(0–10)_. Area under the concentration–time curve was determined only up to 10 h post-dose because of limited availability of data at later timepoints. Dose-normalised AUC for PD 0332991 after administration of 100–225 mg QD demonstrated dose proportionality (Supplementary Figure 1C).

Following repeated daily dosing to steady state (based on samples taken from the 200-mg QD cohort), PD 0332991 was absorbed with a median *T*_max_ of 4.2 h. The mean PD 0332991 *V*_z_/*F* was 3241 l, which is significantly greater than that of total body water (42 l), suggesting that PD 0332991 penetrates extensively into peripheral tissues. The PD 0332991 was eliminated slowly with a mean *t*_1/2_ of 26.7 h; the mean CL/F was 88.5 l per hour. Following repeated dosing, PD 0332991 accumulated with a median *R*_ac_ of 2.4, which is consistent with the *t*_1/2_.

### Pharmacodynamics

**Figure 1:**
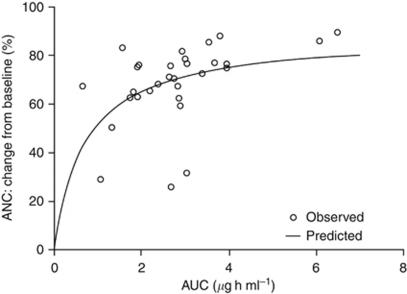
Pharmacodynamic modelling: percentage change in neutrophil count *vs* individual AUC during cycle 1. ANC, absolute neutrophil count; AUC, area under the plasma concentration–time curve.

A nadir in both ANC and levels of platelets was observed at the end of the dosing period in cycle 1 (Supplementary Figure 2), which was followed by a rebound in levels of both ANC and platelets during the off-drug period that continued up to day 8 of cycle 2. The ANC did not return to baseline levels, but platelet levels recovered such that baseline levels were exceeded. From day 9 of cycle 2, ANC and platelet levels declined to the end of the cycle 2 dosing period. Changes from baseline during cycle 1 in levels of ANC and platelets *vs* individual AUC for 30 patients were assessed. For ANC (Figure 1), *E*_max_ was estimated to be 88.6% (s.e. 11.2; 12.6% coefficient of variation (CV) with an EC_50_ of 716 ng h ml^−1^ (s.e. 400; 56.9% CV). For platelets (data not shown), *E*_max_ was estimated to be 92.2% (s.e. 16.9; 18.3% CV) and EC_50_ was estimated to be 1370 ng h ml^−1^ (s.e. 720; 52.9% CV).

## Discussion

This paper presents data from the first-in-human, phase I dose-escalation study of the potent and selective CDK4/6 inhibitor, PD 0332991. Dosing ranged from 100 to 225 mg QD for 2 weeks, followed by 1 week off treatment (Schedule 2/1).

A total of six patients in the overall study population experienced DLT. Of these, four (one patient in the dose-escalation cohort and three patients in the expansion cohort) received a dose of 200 mg QD, which was identified as the MTD. Dose-limiting toxicities consisted exclusively of myelosuppression (neutropenia with or without thrombocytopenia), leading to a delay in initiation of cycle 2 dosing. Overall, myelosuppression was common, with grade 1/2 leukopenia and thrombocytopenia seen in 58–67% of patients, grade 3/4 leukopenia and neutropenia in ∼20–25% of patients, and grade 3/4 lymphopenia in 36% of patients. Preliminary pharmacodynamic modelling suggested that myelosuppression was related to PD 0332991 exposure. Indeed, based on preclinical data, myelosupression was an expected toxicity and is consistent with a mechanism of action that targets the cell cycle (Lin *et al*, 2009). Of note, the lymphopenic toxicity of the CDK inhibitor flavopiridol (see below) resulted in this agent being tested for the treatment of chronic lymphocytic leukaemia, for which it has demonstrated promising activity.

Treatment-related, non-haematological toxicity was generally mild to moderate in intensity with only five grade 3 AEs reported overall and no treatment-related grade 4 toxicity. The most common non-haematological AEs included fatigue and gastrointestinal toxicities consisting primarily of nausea, diarrhoea and constipation, with some patients also experiencing vomiting. Similar AE profiles have been reported for other CDK inhibitors targeting the G1/S checkpoint (Thomas *et al*, 2002; Dittrich *et al*, 2003; Mahadevan *et al*, 2008; Shapiro *et al*, 2008; Tibes *et al*, 2008).

Pharmacokinetics analyses using a non-compartmental model suggest that PD 0332991 exposure increases in a dose-proportional model over the dose range explored in this study, although there was low to moderate inter-patient variability. Population PK studies are ongoing. As anticipated from preclinical data, the half-life of PD 0332991 was relatively long, at 26.7 h, resulting in its accumulation following repeated dosing. Extensive penetration of peripheral tissue was suggested by the large *V*_z_/*F* value (3241 l). Further studies are needed to define precisely when steady state is reached and to characterise the extent of washout in the 1 week off treatment embedded within each cycle.

An exploratory analysis of ANC and platelet levels during the first two treatment cycles showed that levels of both neutrophils and platelets decreased during PD 0332991 treatment, a pharmacodynamic observation that is consistent with CDK inhibition in rapidly dividing cell types. The exploratory analysis demonstrated that the observed nadirs in percentage decreases from baseline were equivalent in each cycle and, importantly, decreases in cell numbers were not cumulative. The lack of cumulative effect indicated a saturable effect of the drug on both ANC and platelets. Furthermore, a PK/pharmacodynamic relationship was established between change in ANC and platelets levels *vs* plasma exposure using a simple *E*_max_ model, with increasing exposures resulting in a decrease from baseline for both ANC and platelets that was saturable. During the 1-week off-drug period, the extent of recovery of neutrophils and platelets was dependent on the cell type. It is possible that multiple factors (including system-based (i.e., cell type) and drug-based) underlie in the overall changes observed following PD 0332991 treatment. More mechanistic population PK/pharmacodynamic modelling, based on a previously described semi-mechanistic physiological model developed to describe the neutropenic effects of cytotoxic agents such as docetaxel, etoposide, paclitaxel and irinotecan (Friberg *et al*, 2002), is currently underway to investigate further the changes in ANC and platelet levels associated with PD 0332991 treatment. It is possible that the use of ANC or platelet modelling may have utility as surrogate pharmacodynamic markers of PD 0332991 activity.

The PD 0332991 showed preliminary evidence of antitumour activity in this study, with one PR (Vaughn *et al*, 2009) and ∼30% of patients exhibiting disease stabilisation, which in three cases was prolonged beyond 10 cycles of treatment. The patient numbers were too small from which to draw conclusions about differences in response between cohorts or within any individual tumour type. The one PR shows that, like certain other cytostatic agents such as tamoxifen, PD 0332991 is capable of both cytostatic and cytotoxic activity in the clinic, as suggested by results from some preclinical models (Fry *et al*, 2004).

Several other CDK inhibitors are in development (Dickson and Schwartz, 2009), including flavopiridol, which has activity against CDK4/6. As previously reported, the activity of PD 0332991 in one of the patients with testicular cancer is striking (Vaughn *et al*, 2009). Flavopiridol has also been shown to be active in patients with germ-cell tumours, especially in combination with oxaliplatin-based chemotherapy (Rathkopf *et al*, 2009). Therefore, CDK inhibitors, particularly those that target CDK4/6, may offer promise for patients with this particular disease.

In summary, PD 0332991 given on Schedule 2/1 is generally well tolerated by patients with advanced solid tumours, with the DLT being myelosuppression. The MTD on this schedule was 200 mg QD, and this dose is recommended for further study in phase II. Phase II studies of PD 0332991 are ongoing/planned in patients with multiple myeloma (ClinicalTrials.gov NCT00555906), breast cancer (ClinicalTrials.gov NCT00721409) and refractory solid tumours (ClinicalTrials.gov NCT01037790). In addition, CDK4 has been shown to be amplified in patients with liposarcoma, which may explain the unusual degree of SD observed in this clinical trial (Singer *et al*, 2007). In view of this, a phase II study of this agent in CDK4-amplified, Rb-positive liposarcoma is now underway (ClinicalTrials.gov NCT01209598).

## Figures and Tables

**Table 1:** Patient baseline characteristics (*N*=33)

**Parameter**	
Age in years, median (range)	63 (35–78)
Male/female, *n* (%)	16/17 (48.5/51.5)
	
*ECOG PS, n (%)*	
0	17 (51.5)
1	11 (33.3)
2	5 (15.2)
	
*Primary diagnosis, n (%)*	
Liposarcoma	7 (21.2)
Colon cancer	6 (18.2)
Melanoma	4 (12.1)
Other	16 (48.5)
	
*Previous therapy, n (%)*	
Any	32 (97.0)
Previous surgery, n (%)	29 (90.6)
Previous radiotherapy, n (%)	14 (43.8)
Previous chemotherapy, n (%)	27 (84.4)
1 regimen	6 (22.2)
2 regimens	5 (18.5)
3 regimens	8 (29.6)
>3 regimens^a^	8 (29.6)
Hormonal	2 (6.3)
Immunotherapy/biological	5 (15.6)

Abbreviation: ECOG PS=Eastern Cooperative Oncology Group performance status.

a Patients who had received >3 previous chemotherapy regimens were documented as protocol deviations.

**Table 2:** Incidence of treatment-emergent neutropenia during cycle 1

		**Neutropenia, NCICTCAE grade (*n*)**				
**Dose QD**	**Patients, *n***	**Grade 0**	**Grade 1**	**Grade 2**	**Grade 3**	**Grade 4**
100 mg	3	0	0	0	0	0
150 mg	4	0	0	0	0	0
200 mg	20	11	0	3	6	0
225 mg	6	3	0	0	2	1

Abbreviations: QD=once daily; NCICTCAE=National Cancer Institute Common Terminology Criteria for Adverse Events.

**Table 3:** Treatment-related non-haematological adverse events^a^ during and after cycle one occurring in ⩾5% of patients for all cohorts combined (*N*=33)

	**Cycle 1, *n* (%)**		**After cycle 1, *n* (%)**	
**Adverse event**	**All grades**	**Grade 3**	**All grades**	**Grade 3**
Fatigue	11 (33)	0	10 (30)	1 (3)
Nausea	10 (30)	0	5 (15)	1 (3)
Diarrhoea	6 (18)	0	10 (30)	0
Constipation	4 (12)	0	3 (9)	0
Rash	4 (12)	0	2 (6)	0
Vomiting	2 (6)	0	2 (6)	1 (3)
Peripheral oedema	3 (9)	0	2 (6)	0
Dyspnea	3 (9)	0	1 (3)	0
Anorexia	1 (3)	0	3 (9)	0
Pyrexia	1 (3)	0	2 (6)	0
Epistaxis	0	0	4 (12)	0
Flatulence	0	0	3 (9)	0
Mucosal inflammation	0	0	3 (9)	0
Chills	0	0	2 (6)	0
Muscular weakness	0	0	2 (6)	0
Cough	0	0	2 (6)	0
Alopecia	0	0	2 (6)	0

a Adverse events were defined as treatment-related based on having a relationship to study drug reported as ‘yes’ or ‘unknown’. No treatment-related, grade 4/5 adverse events were reported.

**Table 4:** Haematological toxicity by maximum toxicity grade among patients whose toxicity worsened relative to baseline across all cohorts and all cycles (*N*=33)

**Haematological toxicity**	**Grade 1/2, *n* (%)**	**Grade 3/4, *n* (%)**
Anaemia	21 (64)	1 (3)
Leukopenia	22 (67)	7 (21)
Neutropenia	14 (42)	8 (24)
Lymphopenia	8 (24)	12 (36)
Thrombocytopenia	19 (58)	3 (9)

**Table 5:** Mean and median plasma pharmacokinetic parameters for PD 0332991 by dose on days 1 and 8

	**100 mg QD**		**150 mg QD**		**200 mg QD**		**225 mg QD**	
**Parameter**	**Day 1 (*n*=3)**	**Day 8 (*n*=3)**	**Day 1 (*n*=4)**	**Day 8 (*n*=3)**	**Day 1 (*n*=20)**	**Day 8 (*n*=8)**	**Day 1 (*n*=6)**	**Day 8 (*n*=6)**
*C*_max_ (ng ml^−1^), mean (%CV)	44 (72)	58 (43)	78 (22)	194 (41)	81 (35)	174 (17)	104 (58)	186 (64)
*T*_max_ (h), median (range)	4.0 (2.0–10.0)	4.0 (4.0–7.0)	6.0 (4.0–10.0)	7.0 (7.0–10.0)	5.7 (1.0–10.2)	4.0 (2.0–7.0)	4.0 (4.0–7.0)	4.5 (1.0–7.0)
AUC_(0–10)_^a^ (ng h ml^−1^), mean (%CV)	333 (NA)	433 (NA)	622 (NA)	1599 (35)	525 (36)	1395 (23)	718 (55)	1491 (64)

Abbreviations: QD=once daily; C_max_=maximum observed plasma concentration; CV=coefficient of variation; T_max_=time-to-first occurrence of C_max_; AUC_(0–10)_=area under the plasma concentration–time curve from 0 to 10 h; NA=not applicable.

a The number of patients on day 1 for the 100-mg, 150-mg and 200-mg cohorts was 2, 2 and 19, respectively, and on day 8 for the 100-mg cohort was 2.

## Appendix

O’Dwyer PJ, LoRusso P, DeMichele A, Gupta V, Barbi A, Dials H, Chen I, Courtney R, Wilner K, Schwartz GK (2007) A phase I dose-escalation trial of a daily oral CDK 4/6 inhibitor PD 0332991. *J Clin Oncol* 25(18S): 150s (abstr 3550).

Vaughn DJ, Flaherty K, Lal P, Gallagher M, O’Dwyer PJ, Wilner K, Chen I, Schwartz GK (2009) Treatment of growing teratoma syndrome. *N Engl J Med* 360(4): 423–424.

Shaik MN, O’Dwyer P, LoRusso PM, Schwartz G, Wilner K, Courtney R (2010) Population pharmacokinetic/pharmacodynamic evaluation of the effect of PD 0332991 on QTc in patients with advanced cancers. Presented at the American Society for Clinical Pharmacology and Therapeutics 2010 Annual Meeting, March 17–20, Atlanta, GA, USA.

Tortorici MA, O’Dwyer P, LoRusso P, Schwartz G, Wilner K, Ruiz-Garcia A, Courtney R (2010) Population pharmacokinetics of PD 0332991 in patients with advanced cancers. Presented at the American Society for Clinical Pharmacology and Therapeutics 2010 Annual Meeting, March 17–20, Atlanta, GA, USA.
