# Supplementary material for: Phase I study of PD 0332991, a cyclin-dependent kinase inhibitor, administered in 3-week cycles (Schedule 2/1)
Source: Br J Cancer. 2011 May 24;104(12):1862–8. doi: 10.1038/bjc.2011.177 (PMC3111206; doi:10.1038/bjc.2011.177)

FIGURE LEGENDS (supplementary online materials)

Supplementary Figure 1. Linear plot of the median plasma concentration–time profile on (A) day 1 and (B) day 8, by dose level; and (C) dose-normalised AUC following a single dose of PD 0332991 as a function of administered dose. QD, once daily; AUC, area under the plasma concentration–time curve.

Supplementary Figure 2. Changes from baseline for (A) neutrophils (ANC)^a^ and (B) platelets^a^ over the first two treatment cycles on the 2/1 dosing schedule. ^a^Includes data that have not been adjusted for treatment delays during cycle 2 in some patients. ANC, absolute neutrophil count.

SUPPLEMENTARY FIGURES

Supplementary Figure 1.


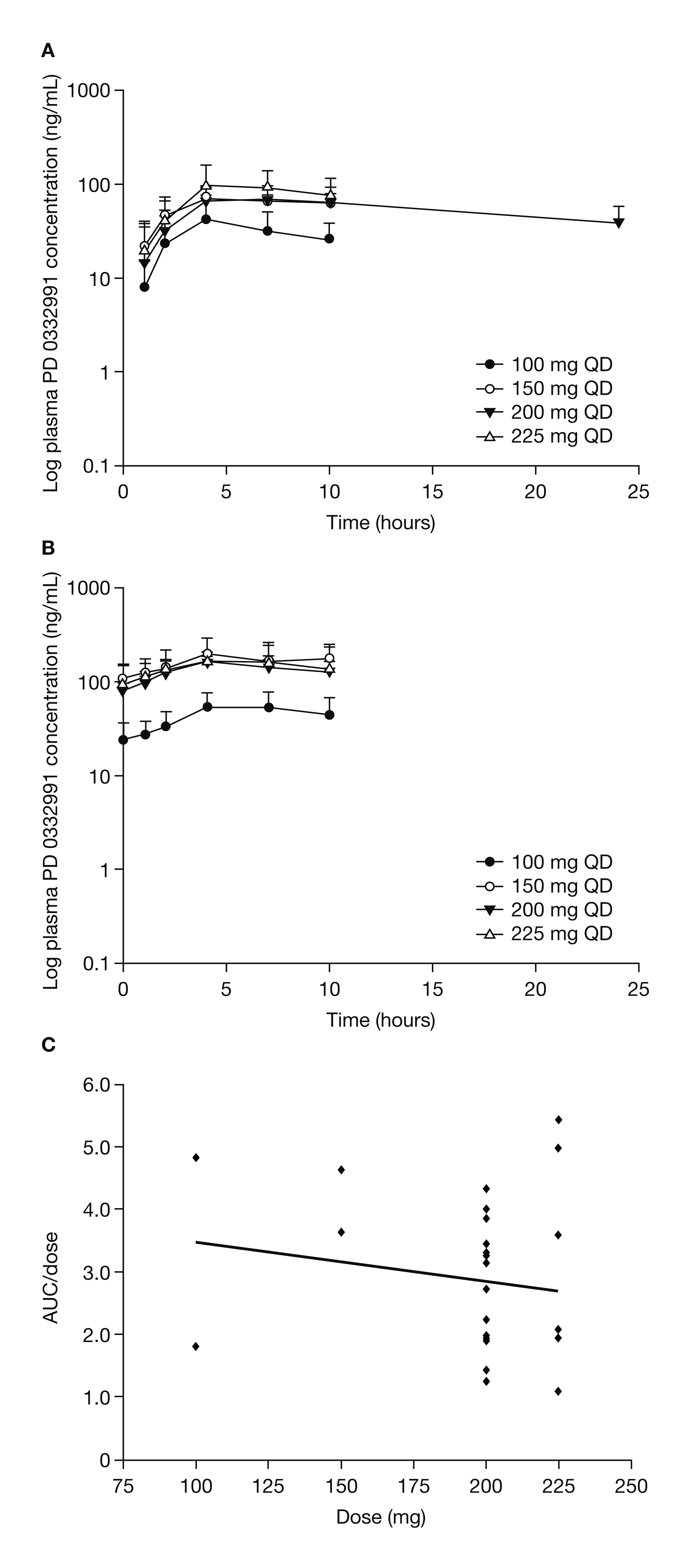


Supplementary Figure 2.


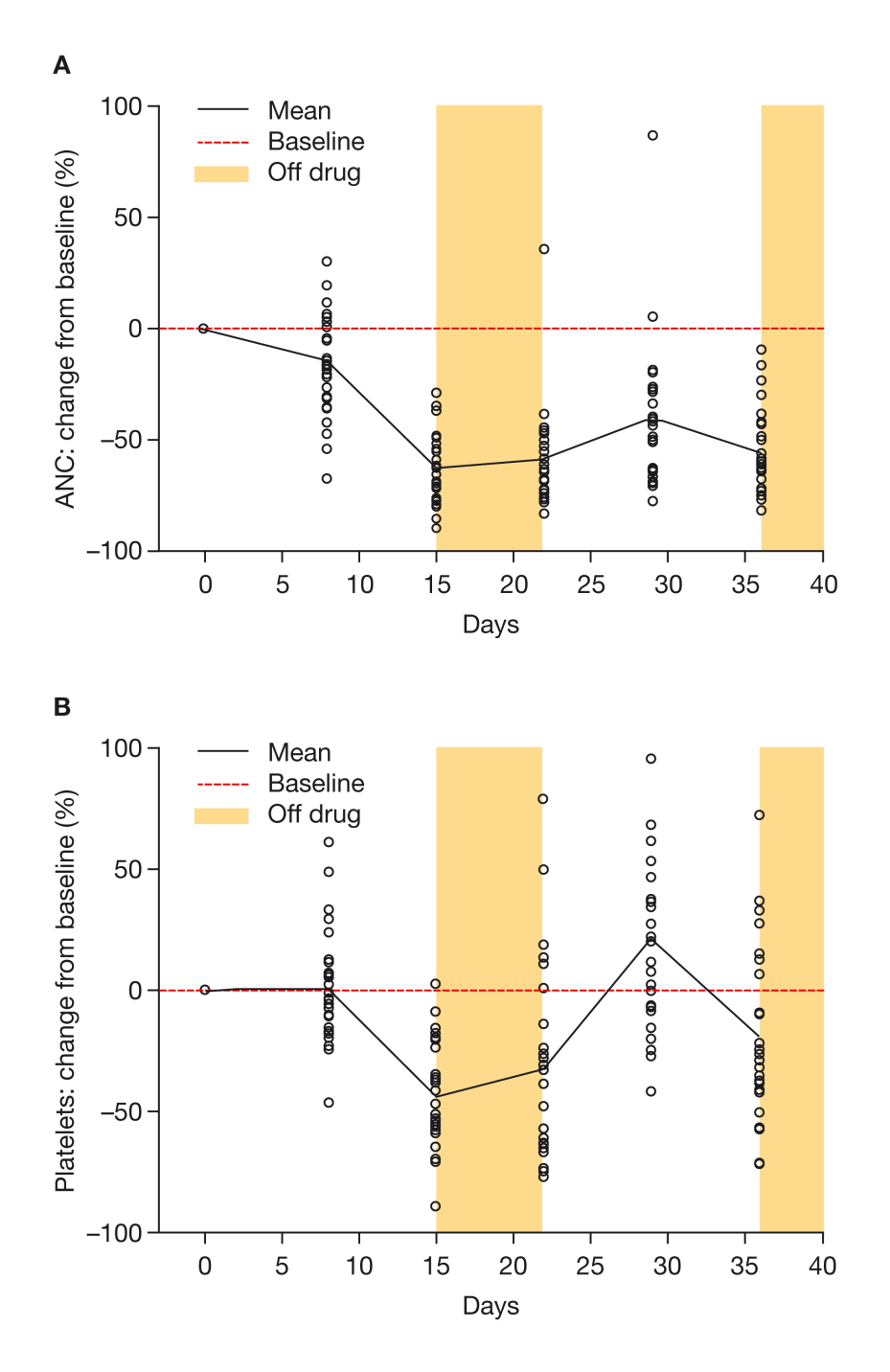

Supplement: Supplementary Figures 1 and 2 [file bjc2011177x1.doc]
